# Improving prediction of solar radiation using Cheetah Optimizer and Random Forest

**DOI:** 10.1371/journal.pone.0314391

**Published:** 2024-12-20

**Authors:** Ibrahim Al-Shourbaji, Pramod H. Kachare, Abdoh Jabbari, Raimund Kirner, Digambar Puri, Mostafa Mehanawi, Abdalla Alameen

**Affiliations:** 1 Department of Electrical and Electronics Engineering, Jazan University, Jazan, Saudi Arabia; 2 Department of Computer Science, University of Hertfordshire, Hatfield, United Kingdom; 3 Department of Electronics & Telecomm, Engineering, Ramrao Adik Institute of Technology, Navi Mumbai, Maharashtra, India; 4 Department of Computer Engineering and Information, Prince Sattam Bin Abdulaziz University, Wadi Alddawasir, Saudi Arabia; Xi’an Jiaotong University, CHINA

## Abstract

In the contemporary context of a burgeoning energy crisis, the accurate and dependable prediction of Solar Radiation (SR) has emerged as an indispensable component within thermal systems to facilitate renewable energy generation. Machine Learning (ML) models have gained widespread recognition for their precision and computational efficiency in addressing SR prediction challenges. Consequently, this paper introduces an innovative SR prediction model, denoted as the Cheetah Optimizer-Random Forest (CO-RF) model. The CO component plays a pivotal role in selecting the most informative features for hourly SR forecasting, subsequently serving as inputs to the RF model. The efficacy of the developed CO-RF model is rigorously assessed using two publicly available SR datasets. Evaluation metrics encompassing Mean Absolute Error (MAE), Mean Squared Error (MSE), and coefficient of determination (*R*^2^) are employed to validate its performance. Quantitative analysis demonstrates that the CO-RF model surpasses other techniques, Logistic Regression (LR), Support Vector Machine (SVM), Artificial Neural Network, and standalone Random Forest (RF), both in the training and testing phases of SR prediction. The proposed CO-RF model outperforms others, achieving a low MAE of 0.0365, MSE of 0.0074, and an *R*^2^ of 0.9251 on the first dataset, and an MAE of 0.0469, MSE of 0.0032, and *R*^2^ of 0.9868 on the second dataset, demonstrating significant error reduction.

## Introduction

Over the decade, industrial modernization has constantly increased the energy demand of our economy, while the energy supply amount is limited. The continually diminishing conventional resources like fossil fuels, petroleum, coal, and natural gas have further increased the demand-supply gap, commonly known as the energy crisis. In search of green and sustainable energy, efforts are encouraged to use renewable resources like solar, wind, water, biomass, and geothermal. These renewable resources reduce environmental pollution and stress on conventional resources [1–4].

Solar Radiation (SR) is a readily and abundantly available renewable resource in most parts of the world. In recent years, SR prediction has received significant attention in academia [5–8]. Several ML models have been reported to predict SR [9]. Compared support vector machine (SVM) and Extreme Gradient Boosting (XGBoost) for SR prediction. Comparative evaluation using prediction accuracy, stability, and computational time showed that XGBoost outperformed SVM for estimating SR. In [10] work, the authors assessed the ability of kernel-based nonlinear extension of Arps decline and Gradient Boosting (GB) models with categorical features to support daily global SR prediction. Both models were reported to be suitable for SR. In another work [11], bagging and boosting ensemble models improve the performance of SVM, decision trees, and artificial neaural network (ANN) models for hourly SR prediction. The results demonstrated that decision trees improved SR prediction than the other used models.

RF technique is also widely applied for energy systems and SR prediction [12–15], used Firefly (FF) algorithm to choose the best number of leaves per tree for the RF. The outcomes showed that the combined model outperformed the conventional Random Forest (RF), and ANN. Also, optimizing the ANN regarding the accuracy and execution speed also enhances the overall performance [16, 17]. In another work [18], Particle Swarm Optimization (PSO) was utilized to optimize RF parameters for SR prediction. The results showed that PSO-RF achieved better SR prediction than the decision trees, RF, and ANN. In [19] work, RF, random tree, reduced error pruning trees, and hybrid random tree reduce model are compared for daily SR prediction using four locations located in Burkina Faso. The results confirmed that RF is a promising approach for predicting SR accurately. In another comparison study [20], RF and three other tree-based machine learning (ML) models for SR prediction in India are compared. The results indicated that RF outperformed the other models used in their study. Benali *et al*. [21], smart persistence, ANN, and RF are compared to predict global horizontal, diffuse horizontal, and beam normal components and SR in France. The results showed that RF predicted all components more effectively than other methods. In another recent work, [22], the authors compared seven ML models for SR prediction. The obtained results showed that RF performed better than the other compared methods.

A hybrid ML technique is proposed for hour global SR prediction [23]. The developed approach relied on a Convolutional Neural Network, Non-parametric Gaussian Process Regression (GPR), Least square SVM (LS-SVM), and Extreme Learning Machine (ELM). Experimental results showed that the proposed approach attained better accuracy than other popular ML models. In another work, Kuhe *et al*. [24], the authors attempt to improve the prediction accuracy of SR using ANN’s ensemble. They used Makurdi city in Nigeria data as a case study to evaluate the model. The results showed that the proposed model achieves great accuracy. Pang *et al*. [25], showed that the Recurrent Neural Network (RNN) can achieve higher accuracy for SR than the ANN model. In [26] work, a system to predict SR using Neural Networks is proposed. The authors used meteorological data from five different cities in Bangladesh. They used RNN, long short term memory (LSTM), and Gated Recurrent Unit (GRU) models to train the meteorological data. Among the used models, the GRU performed the best. Geshnigani *et al*. [27], improved conventional Multiple Llinear Regression, Multilayer Perceptron Neural Network, and Adaptive Neuro-Fuzzy Inference System (ANFIS) using optimization algorithms. They confirmed that the ANFIS model is effectively enhanced when optimization methods are used. Although deep learning-based models have shown success in a variety of areas over the past few years, these models require large datasets or need optimization of model parameters and latency to achieve generalization [28–30].

The metaheuristic models have shown good performance in variation applications [31, 32]. Wu *et al*. [33], used Ant Colony Optimization (ACO), Cuckoo Search (CS) and Grey Wolf Optimization (GWO) algorithms to optimize the SVM model for SR prediction. Among the hybrid models, GWO-SVM had the best results. In the work of Goliatt and Yaseen [34], a computation intelligent model based on the hybridization of Covariance Matrix Adaptive Evolution Strategies (CMAES) with XGBoost and Multi-Adaptive Regression Splines (MARS) models for building robust predictive models for daily SR prediction is developed. Results showed that the developed approach improves prediction accuracy. Gupta *et al*. [35], merged Variance Inflation Factor with Mutual Information (VIFMI) as a feature selection method and then the most important features are used as inputs to a Stack Ensemble Extra Tress (SEET) model for estimating SR. Results showed that the developed ensemble model effectively reduced prediction error.

Due to the rapid deployment of solar energy techniques, SR has become common in recent years, and several ML models are widely used to predict SR. However, ML techniques can encounter challenges regarding input data, which can affect the efficacy of these models Banadkooki *et al*. [36], choosing relevant features is critical for building a learning model, as the input feature set influences it. This plays a pivotal role in maintaining high classification accuracy and reducing the dimensionality of datasets. According to the author’s best knowledge, well-known methods like PSO, ACO, GWO and FF perform well either in local or global searches, and they have poor performance in balancing between local and global searches. CO is a new population-based algorithm introduced in 2022 by Akbari [37] as a powerful method for mimicking specific cheetahs’ hunting strategies. CO shows a better balance between local and global search ability. Hence, in the present work. A new hybridization approach has been developed for more reliable and accurate SR predictions. For this purpose, the hybrid of CO and RF (CO-RF) is employed for predicting SR. The key contributions of this paper can be summarised as follows:

- Proposing an efficient prediction model, CO-RF, for SR by integrating optimization algorithm rather than the use of existing ML models alone.
- Evaluating the developed CO-RF model on two publicly available datasets and three evaluation metrics: mean absolute error (MAE), mean square error (MSE), and *R*^2^.
- Investigating CO-RF’s effectiveness in performing SR prediction during training and testing phases and comparing its efficiency to other models before and after using optimization algorithms.

The rest of this paper is structured as follows: Section 2 describes the datasets and methods used and the developed CO-RF model for SR prediction. The statistical metrics to evaluate the ML models are presented in section 3. Comparative analysis of the models is presented in section 4, and section 5 concludes the paper.

## Materials and methods

Worldwide, solar energy is an increasingly important renewable energy source. Accurate prediction of SR is crucial for the efficient design and operation of solar power systems. A publicly available SR dataset for measurements is necessary to address this need. In this work, two such datasets are used for experimental evaluations.

### Dataset-1

HI-SEAS weather station collected the SR dataset and made it accessible to the scientific community as part of the NASA hackathon task NASA (2023) [38]. The dataset encompasses various meteorological attributes, enabling researchers to examine the intricate relationships between various environmental factors and SR. It comprises eleven distinct features that provide a comprehensive insight into the environmental conditions that influence SR patterns. In preparation for analysis, the UNIX time, a timestamp format, is transformed to facilitate the sorting and organization of recorded measurements. Two critical columns are derived from UNIX time: ‘Month’ and ‘Year,’ enabling temporal analysis of SR trends. A ‘Sun availability’ feature is generated as a difference between sunrise and sunset times, shedding light on the temporal dynamics of sunlight exposure. SR values are scaled to kilo-watts per square meter (kW/m²) to mitigate the impact of large variations in SR values, ultimately enhancing the performance of ML models. The processed dataset comprises 32,686 records and nine distinct features that encapsulate vital meteorological and temporal parameters. A comprehensive statistical analysis of the dataset is given in Table 1.

**Table 1:** Statistical summary of dataset1.

Feature	Unit	Mean	SD	Min	25%	50%	75%	Max
Recording time	UNIX time	1.48E+9	3.00E+6	1.47E+9	1.47E+9	1.48E+9	1.48E+9	1.48E+9
Temperature	Fahrenheit	51.103	6.201	34.000	46.000	50.000	55.000	71.000
Pressure	Hg	30.423	0.055	30.190	30.400	30.430	30.460	30.560
Humidity	%	75.016	25.990	8.000	56.000	85.000	97.000	103.000
Wind direction	Degrees	143.490	83.168	0.090	82.228	147.700	179.310	359.950
Wind speed	m/hr	6.244	3.490	0.000	3.370	5.620	7.870	40.500
Hour	24 hr	11.557	6.912	0.000	6.000	12.000	18.000	23.000
Month	-	10.489	1.235	1.000	10.000	11.000	11.000	12.000
Sun availability	0-12 hr	11.348	0.476	11.000	11.000	11.000	12.000	12.000

### Dataset-2

This dataset is collected and maintained by the King Abdullah City for Atomic and Renewable Energy in Saudi Arabia, provided by the OpenData platform, a government-based repository for open data, for all experimental evaluations [39]. The dataset comprises 1265 records, each characterized by 26 distinct features. These features encapsulate various aspects of solar power generation and meteorological conditions. The data is collected from 41 solar power facilities in Saudi Arabia, as described in Table 2.

**Table 2:** Distribution of solar radiation records across Saudi Arabia.

Location	No. of records	Location	No. of records
Al-Aflaaj Technical Institute	37	Najran University	31
Al-Baha University	20	Prince Sattam Bin Abdulaziz University	36
Al-Dawadmi College of Technology	36	Princess Norah University	6
Al-Hanakiyah Technical Institute	35	Qassim University	38
Al-Jouf College of Technology	20	Rania Technical Institute	20
Al-Qunfudhah Technical Institute	35	Royal-Commission of Jubail & Yanbu	20
Al-Uyaynah Research Station	42	Saline Water Conversion Corp., Al-Khafji	20
Al-Wajh Technical Institute	34	Saline Water Conversion Corp., Farasan	23
Arar Technical Institute	20	Saline Water Conversion Corp., Hagl	36
Duba Technical Institute	36	Saline Water Conversion Corp., Jubail	35
Hafar Al-Batin Technical College	34	Saline Water Conversion Corp., Umluj	35
Hail College of Technology	19	Shaqra University	36
Jazan University	21	Sharurah Technical Institute	35
K.A.CARE, Olaya	42	Tabuk University	34
K.A.CARE, City Site	42	Taibah University	17
King Abdulaziz University, Osfan	34	Taif University	38
KAU of Science & Technology	38	Timaa Technical Institute	36
King Fahd University of Petroleum Minerals	38	Umm Al-Qura University	17
King Faisal University	38	University of Dammam	38
King Saud University	21	Wadi Addawasir College of Technology	36
Majmaah University	36	**Total**	**1265**

Preliminary data analysis revealed that the feature ‘Wind Speed at 3m (SD) Uncertainty (m/s)’ has missing values for all site locations. Hence, this feature is dropped. Also, four records are dropped due to missing solar radiation values. To enhance the utility of the dataset, several preprocessing steps are undertaken. The average values of the corresponding feature impute the missing values in all remaining records. After processing the missing values, each feature is normalized independently to have zero mean and unity standard deviation. The solar radiation values represented by the Global Horizontal Irradiance (GHI), is normalized within the range [-1, +1]. This normalization accounts for variations in power production among different solar stations, ensuring that the dataset maintains consistency and is suitable for machine-learning applications. The processed dataset has 1262 records and 22 features. The GHI features have a mean value of 0.117 and a Standard Deviation (SD) of 0.492. A comprehensive statistics of the dataset is described in Table 3.

**Table 3:** Statistical summary of dataset2.

Feature	Unit	Min	25%	50%	75%	Max
Air Temperature	Celsius	-2.546	-0.798	0.133	0.838	1.810
Air Temperature Uncertainty	Celsius	-0.040	-0.040	-0.040	-0.040	25.100
Wind Direction at 3m	Degree	-1.542	-0.948	0.000	1.023	1.447
Wind Direction at 3m Uncertainty	Degree	-13.497	0.164	0.164	0.164	2.896
Wind Speed at 3m.	m/s	-3.271	-0.676	-0.028	0.405	5.054
Wind Speed at 3m Uncertainty.	m/s	-1.549	-1.549	0.678	0.678	0.678
Wind Speed at 3m.	m/s	-3.469	-0.731	-0.099	0.532	4.955
Diffuse Horizontal Irradiance (DHI)	Wh/*m*^2^	-1.864	-0.867	-0.055	0.735	2.692
DHI Uncertainty	Wh/*m*^2^	-1.629	-0.804	-0.118	0.688	8.167
DHI Standard Deviation	Wh/*m*^2^	-2.396	-0.626	0.000	0.507	4.263
Direct Normal Irradiation (DNI)	Wh/*m*^2^	-2.900	-0.715	-0.015	0.588	3.089
DNI Uncertainty	Wh/*m*^2^	-1.767	-0.871	0.139	0.525	6.931
DNI Standard Deviation	Wh/*m*^2^	-2.659	-0.613	0.000	0.566	3.003
Global Horizontal Irradiance (GHI)	Wh/*m*^2^	-1.000	-0.272	0.154	0.540	1.000
GHI Uncertainty	Wh/*m*^2^	-0.729	-0.368	-0.032	0.186	19.933
Standard Deviation GHI	Wh/*m*^2^	-1.790	-0.725	0.000	0.432	4.114
Peak Wind Speed at 3m.	m/s	-3.978	-0.731	-0.123	0.486	4.746
Peak Wind Speed at 3m Uncertainty	m/s	-5.274	0.117	0.117	0.117	5.507
Relative Humidity	%	-1.494	-0.964	-0.072	0.773	2.269
Relative Humidity Uncertainty	%	-8.375	-0.049	-0.049	-0.049	20.765
Barometric Pressure	hPa	-2.657	-0.675	-0.258	1.043	1.431
Barometric Pressure Uncertainty	hPa	-2.459	-0.783	-0.364	0.892	4.662

### Cheetah Optimizer (CO)

The CO is a recent metaheuristic method proposed by [37]. This method mimics the behaviours of a cheetah. In this algorithm. a cheetah starts the hunting process by scanning its environment (defined as search space) to detect probable prey. Depending on the distance between the cheetah and the prey, the cheetah may hold the position and wait for the prey to get closer. The rushing and capturing are two stages of the cheetah’s attack. The hunt may be terminated due to reduced energy limits, fast prey feeling, etc. After termination, the cheetah returns home to rest before the next hunt. The pseudo-code of the CO is provided in [37]. The CO chooses one of the following strategies depending on the prey, their condition, search area and distance of the prey:

- *Search strategy*:
  Cheetahs find the probable prey in the environment by scanning the area while sitting/standing or patrolling the area actively. The scanning mode is suitable while walking on the plains for dense and grazing prey. On the other hand, scattered and active prey demand more energy-consuming active mode. The cheetah selects a sequence of these modes during the hunt depending on its own condition, the condition of prey, and area coverage. For *i*^*th*^ cheetah in *j*^*th*^ arrangement, the new position $X_{\left(i,j\right)}^{\left(t+1\right)}$ is calculated based on their current position $X_{i}^{t}$ as follows:
  $$\begin{array}{c} X_{\left(i,j\right)}^{\left(t+1\right)}=X_{i}^{t}+r_{\left(i,j\right)}^{\left(\Delta -1\right)}\cdot a_{\left(i,j\right)}^{t} \end{array}$$ (1)
  Where, t is the hunting time with maximum limit *T*, $r_{\left(i,j\right)}^{\left(\Delta -1\right)}$ is the random number, and $a_{\left(i,j\right)}^{t}$ is step size.
- *Sit and wait strategy*:
  When the prey is in the cheetah’s vision field, any movement may reveal the cheetah’s presence to the prey and allow the prey to escape. To avoid the prey’s escape, the cheetah gets closer to the prey by hiding among the bushes or lying on the ground before the ambush. Therefore, in this mode, the cheetah remains at their position and waits for the prey to come nearer, and this behaviour can be represented as:
  $$\begin{array}{c} X_{\left(i,j\right)}^{\left(t+1\right)}=X_{\left(i,j\right)}^{t} \end{array}$$ (2)
- *Attack strategy*
  Speed and flexibility are two crucial factors in a cheetah’s attack. The cheetah starts the attack by rushing towards the prey at full speed. When the prey starts to flee as soon as it realizes the cheetah’s attack, the cheetah adjusts the path to intervene in the prey. For the current prey position $X_{\left(P,j\right)}^{t}$ in *j*^*th*^ arrangement, the cheetah’s position according to this attack strategy is mathematically defined as:
  $$\begin{array}{c} X_{\left(i,j\right)}^{\left(t+1\right)}=X_{\left(P,j\right)}^{t}+\gamma_{\left(i,j\right)}^{\left(\Delta -1\right)}.b_{\left(i,j\right)}^{t} \end{array}$$ (3)
  where, $\gamma_{\left(i,j\right)}^{\left(\Delta -1\right)}$ and $b_{\left(i,j\right)}^{t}$ are turning and interaction factors of *i*^*th*^ cheetah in *j*^*th*^ arrangement.

The Cheetah Optimizer operates through the following steps:

- Initialization: The algorithm starts by initializing parameters and setting hyper-parameters such as learning rates and momentum terms.
- Gradient Computation: At each iteration, the gradient of the objective function with respect to the parameters is computed.
- Update Step: The optimizer uses a specific update rule, which is influenced by the computed gradient and hyper-parameters, to adjust the parameters.
- Convergence Check: The optimizer checks whether the changes in the objective function or parameters are below a certain threshold to determine if convergence has been reached.
- Parameter Update: If the convergence criteria are not met, the algorithm updates the parameters and repeats the process.

### Random Forest (RF)

Random Forest [40] is a machine learning model that builds an ensemble of decision trees, with each tree trained using a subset of randomly selected examples and features. This diversity in data for each tree reduces overfitting and improves robustness [41]. The final prediction is typically the pooling of predictions from trees in the forest. The ensemble approach generalizes well to new data, making RF popular for various ML problems [42]. RF builds trees in parallel and, hence, is less prone to overfitting, leading to faster training times. RF does not require extensive tuning of hyperparameters due to built-in overfitting controlling mechanisms, like random subsampling of records and features. The primary hyperparameters for tuning in RF include the number of trees in the ensemble and the maximum depth of each tree. In practice, it uses deep trees, and the number of trees is selected based on model performance [43].

**Fig 1:**
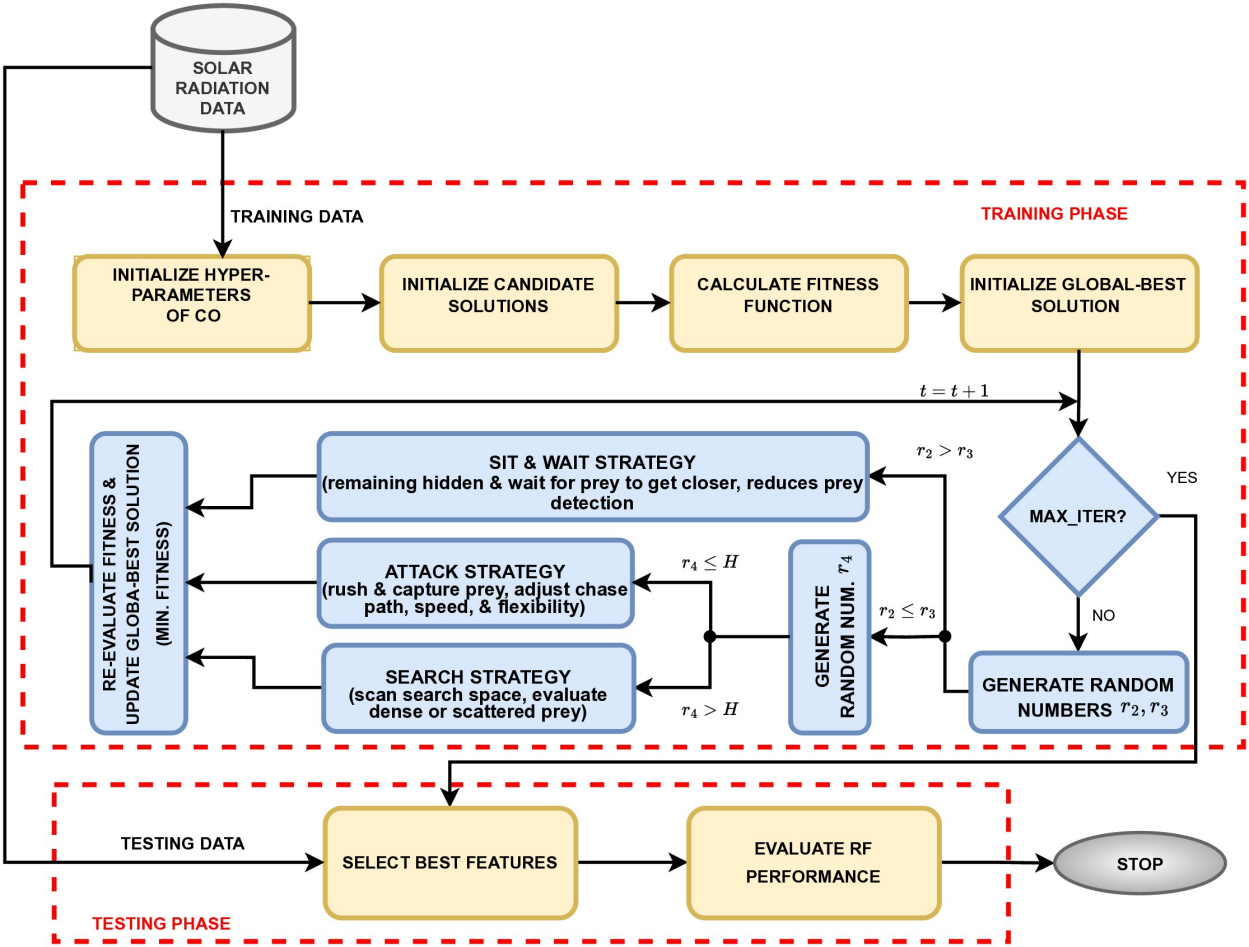
System flow of CO-RF model for SR prediction.

The proposed system for SR prediction uses CO for selecting optimum features and RF for evaluating performance using these optimized features, as shown in system flow in Fig 1. The hyper-parameters model with the number of trees and a maximum depth determined through cross-validation.

## Experiments and results

### Experimental setup

All the ML models are implemented using the Python-based Scikit-Learn library, capitalizing on its robust model development and evaluation capabilities. The experimentation environment employed was a Windows 10 operating system on an Intel i7 processor clocked at 3.13 GHz and 64 GB of RAM. Lower fitness values indicate better feature selection, enhancing the SR prediction models. These methods are: Particle Swarm Optimization (PSO) [44], ACO [45], Reptile Search Algorithm (RSA) [46], Snake Optimizer (SO) [47], and CO, [37]. The standard parameters of these methods are selected empirically and are set: Population size = 20, number of *iterations* = 100, and each of them is executed indecently run ten times mitigate biases towards local minima. The settings of these methods are defined based on their implementations in original works, and they are listed in Table 4.

**Table 4:** Parameters settings.

Algorithm	Parameters
PSO	*c*_1_ = *c*_2_ = 2; *w*_*min*_ = 0.1 *and W*_*max*_ = 0.9
ACO	*τ*_0_ = 1; *p* = 0.95; *α* = 1.2; *β* = 0.5.
RSA	*γ* = 0.9; *θ* = 0.5.
SO	*c*_1_ = 0.5; *c*_2_ = 0.05; *c*_3_ = 2
CO	*n* = 6; *m* = 2.
LR	Regression type = Lasso (L1); Regression coefficient = 1.
SVM	Regularization = 10; kernel = radial basic function; gamma = 0.01.
ANN	Hidden layers = (20, 2); activation = ReLU; solver = Adam, batch_size = 200.
RF	No. of Trees = 200; impurity = gini, max_features = 14; min_samples_split = 5.
CO-RF	Uses the parameters of the CO and RF

### Evaluation metrics

To assess prediction results, three different statistical measures are used for the evaluation of the developed CO-RF and the other ML models. These metrics are selected due to their suitability for SR prediction in previous works [22, 33, 48, 49], and they are defined as follows:

$$\begin{array}{c} MAE=\frac{1}{N}\sum_{i=1}^{N}\left|m_{i}-p_{i}\right| \end{array}$$ (4)

$$\begin{array}{c} MSE=\frac{1}{N}\sum_{i=1}^{N}{\left(m_{i}-p_{i}\right)}^{2} \end{array}$$ (5)

$$\begin{array}{c} R^{2}=1-\frac{\sum_{i}{\left(m_{i}-p_{i}\right)}^{2}}{\sum_{i}{\left(m_{i}-\bar{m}\right)}^{2}} \end{array}$$ (6)

where, *N* is the number of observations, *m*_*i*_ is the ith measured SR value, *p*_*i*_ is the ith SR value predicted by the model and *μ*_*im*_ is the mean of the measured SR values.

### Results and discussion

**Fig 2:**
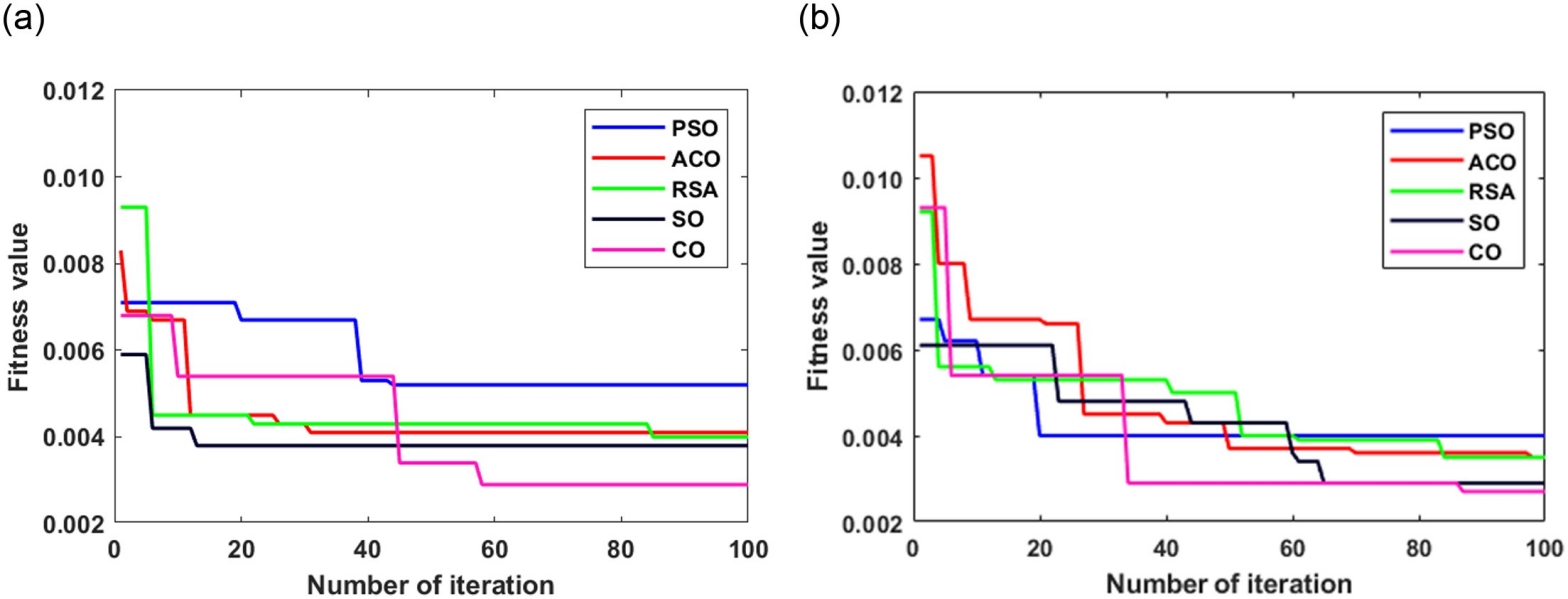
Convergence behavior of different optimization algorithms as feature selectors for SR prediction for (a) dataset-1 and (b) dataset-2.

The performance of the models in predicting SR is rigorously evaluated and provided in this section. The average convergence behavior of these algorithms is presented in Fig 2. In Fig 2a, the CO algorithm achieves a minimum convergence fitness of 0.0029. PSO and ACO also demonstrated strong feature selection capabilities with fitness values of 0.0052 and 0.0041, respectively. CO stood out as the top-performing algorithm for dataset-1. These results provide valuable insights for researchers and practitioners looking to optimize feature selection for SR prediction tasks in similar datasets. As shown in Fig 2b, CO converges at a fitness value of 0.0027 for dataset-2. The fitness values represent the quality of feature selection for enhancing SR prediction. SO also demonstrated strong feature selection abilities, yielding a fitness value of 0.0029. ACO and RSA reported the same fitness value of 0.0035. PSO achieved the worst performance with a 0.0040 fitness value. The marginally higher convergence value in dataset-1 may be attributed to the larger number of records than in dataset-2.

CO reported the smallest fitness values for both datasets, indicating the best performance compared to the other optimization algorithms. Hence, further analysis uses only CO as a feature selection method. CO chooses five salient features from the original nine features in dataset-1 and fourteen out of the original twenty-one features in dataset-2. The selected salient features from both datasets are subsequently employed as inputs for various ML algorithms to assess their capabilities for SR prediction.

The computation time required for either dataset is important to justify the model’s scalability. The overall computation time required by the hybrid model can be divided into three parts: time for feature selection by CO, time for training the ML model using the selected features, and time for performance evaluation. Most of the time required was for feature selection due to the iterative nature of the process. The ML model training time varied over different folds depending upon the number of selected features. The testing performance time had almost insignificant variations across different folds. The overall time for dataset-1 was 30.125 ± 2.536 and for dataset-2 was 10.983 ± 0.843. It was observed that almost 90-93% of the overall time was for feature selection, and approximately 1% of the overall time was for performance evaluation. It must be noted that the feature selection process is required only during the model training. In the testing phase, the most important feature already indicated by the training process can only be calculated further to reduce the solar radiation prediction time than the models using the complete feature set.

#### Predictive performance

The selected features are assessed using MAE, MSE, and *R*^2^ measures. Table 5 compares the predictive performance of the ML models using a complete feature set and with a CO-based selected feature set across both datasets. The models depicted with the prefix ‘CO-’ use features optimized with CO. Each model is validated using 10-fold cross-validation and resulting metric in terms of both mean and SD.

**Table 5:** Comparative performance of ML models with and without CO for SR prediction.

Dataset	Model	MAE	MSE	*R* ^2^
Dataset-1	LR	0.1523 ± 0.0037	0.0389 ± 0.0036	0.6162 ± 0.0167
	SVM	0.0826 ± 0.0115	0.0226 ± 0.0050	0.8163 ± 0.0565
	ANN	0.0593 ± 0.0023	0.0128 ± 0.0015	0.9023 ± 0.0129
	RF	0.0412 ± 0.0016	0.0095 ± 0.0007	0.9165 ± 0.0071
	CO-LR	0.1475 ± 0.0035	0.0382 ± 0.0018	0.6208 ± 0.0176
	CO-SVM	0.0729 ± 0.0118	0.0176 ± 0.0051	0.8337 ± 0.0576
	CO-ANN	0.0439 ± 0.0023	0.0106 ± 0.0015	0.9175 ± 0.0126
	CO-RF	0.0365 ± 0.0015	0.0074 ± 0.0007	0.9251 ± 0.0073
Dataset-2	LR	0.1105 ± 0.0072	0.0102 ± 0.0056	0.8936 ± 0.0165
	SVM	0.0762 ± 0.0069	0.0119 ± 0.0045	0.9510 ± 0.0163
	ANN	0.0745 ± 0.0059	0.0103 ± 0.0017	0.9571 ±0.0080
	RF	0.0469 ± 0.0031	0.0032 ± 0.0004	0.9868 ± 0.0018
	CO-LR	0.0927 ± 0.0069	0.0089 ± 0.0045	0.9093 ± 0.0163
	CO-SVM	0.0737 ± 0.0069	0.0119 ± 0.0045	0.9510 ± 0.0163
	CO-ANN	0.0745 ± 0.0059	0.0103 ± 0.0017	0.9571 ±0.0080
	CO-RF	0.0469 ± 0.0031	0.0032 ± 0.0004	0.9868 ± 0.0018

In the evaluation of dataset-1, a distinct performance variation is observed among SR prediction models. LR exhibited MAE of 0.1523, indicating a reasonable prediction accuracy. However, introducing CO hybridization (CO-LR) slightly improved prediction, and the *R*^2^ value increased to 0.6208. On the other hand, SVM demonstrated superior performance than LR, with MAE of 0.0826, a lower MSE of 0.0226, and a higher *R*^2^ of 0.8163. Applying CO hybridization to SVM (CO-SVM) enhanced its predictive power, resulting in an *R*^2^ of 0.8337. ANN and RF models also exhibited commendable performance. The hybridization of CO (CO-ANN and CO-RF) leads to significant improvements.

In dataset-2, LR achieved an MAE of 0.1105, showing a competitive predictive capability. The CO hybridization (CO-LR) slightly decreased the MAE, and the *R*^2^ value elevated to 0.9093. SVM continued to excel on this dataset, with a mean MAE of 0.0762 and an impressive *R*^2^ of 0.9510. The application of CO hybridization (CO-SVM) maintained high-performance levels, reaffirming its effectiveness. Additionally, ANN and RF models displayed high performance, and the inclusion of CO (CO-ANN and CO-RF) significantly improved their performance.

Dataset 1 showcased model variability, with RF emerging as a standout performer, notably bolstered by CO hybridization. Similarly, dataset-2 demonstrated consistent accuracy across models, with RF again excelling and CO hybridization reaffirming its efficacy. While LR and ANN models showed competitive results, the introduction of CO had a significant impact. To summarize, the dataset-dependent nature of SR prediction models, with CO hybridization potentially enhancing ML-based approaches. Hence, if performed using CO-optimized features, all further analysis is hybridized with four ML models.

#### Comparative performance of CO-optimized ML models

**Fig 3:**
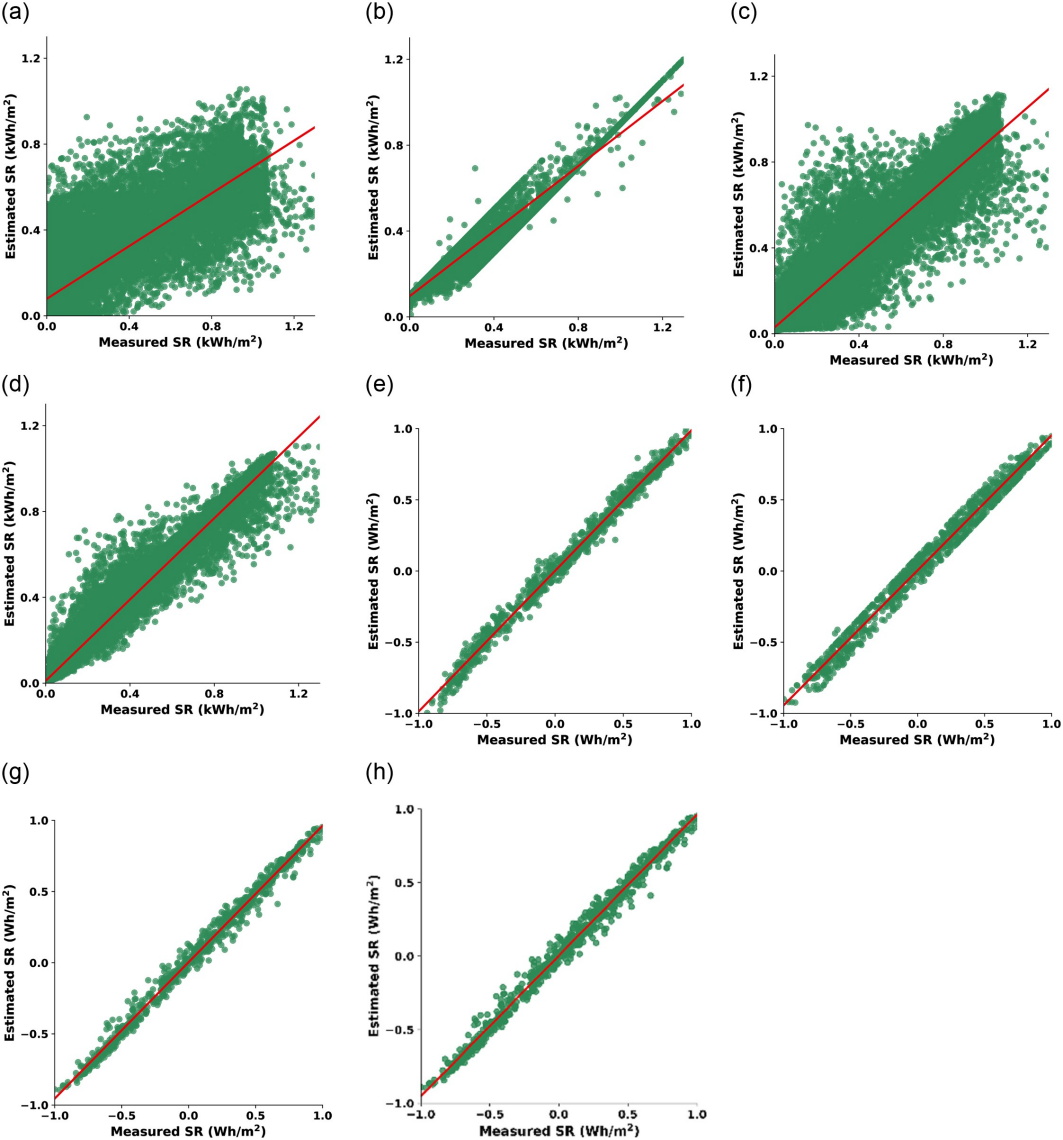
Convergence behavior of CO-optimized ML models using training data of dataset-1 (first row, (a)-(d)) and dataset-2 (second row, (e)-(h)): CO-LR ((a) & (e)), CO-SVM ((b) & (f)), CO-ANN ((c) & (g)), and CO-RF ((d) & (h)).

Visualizations are employed to provide intuitive insights into the CO-based models’ predictive behavior. Fig 3 provides eight scatter plots for essential insights into the performance of four ML models across training data of both SR datasets. These scatter plots employ measured SR values, sourced from the datasets, on the horizontal axis, while the SR estimated by the ML models is plotted on the vertical axis. The ideal scenario in these scatter plots is a perfectly diagonal line where the estimated SR values align precisely with the measured SR values. Such alignment represents accurate predictions by the models. Consequently, the further the data points deviate from the diagonal line, the less accurate the model’s predictions become.

**Fig 4:**
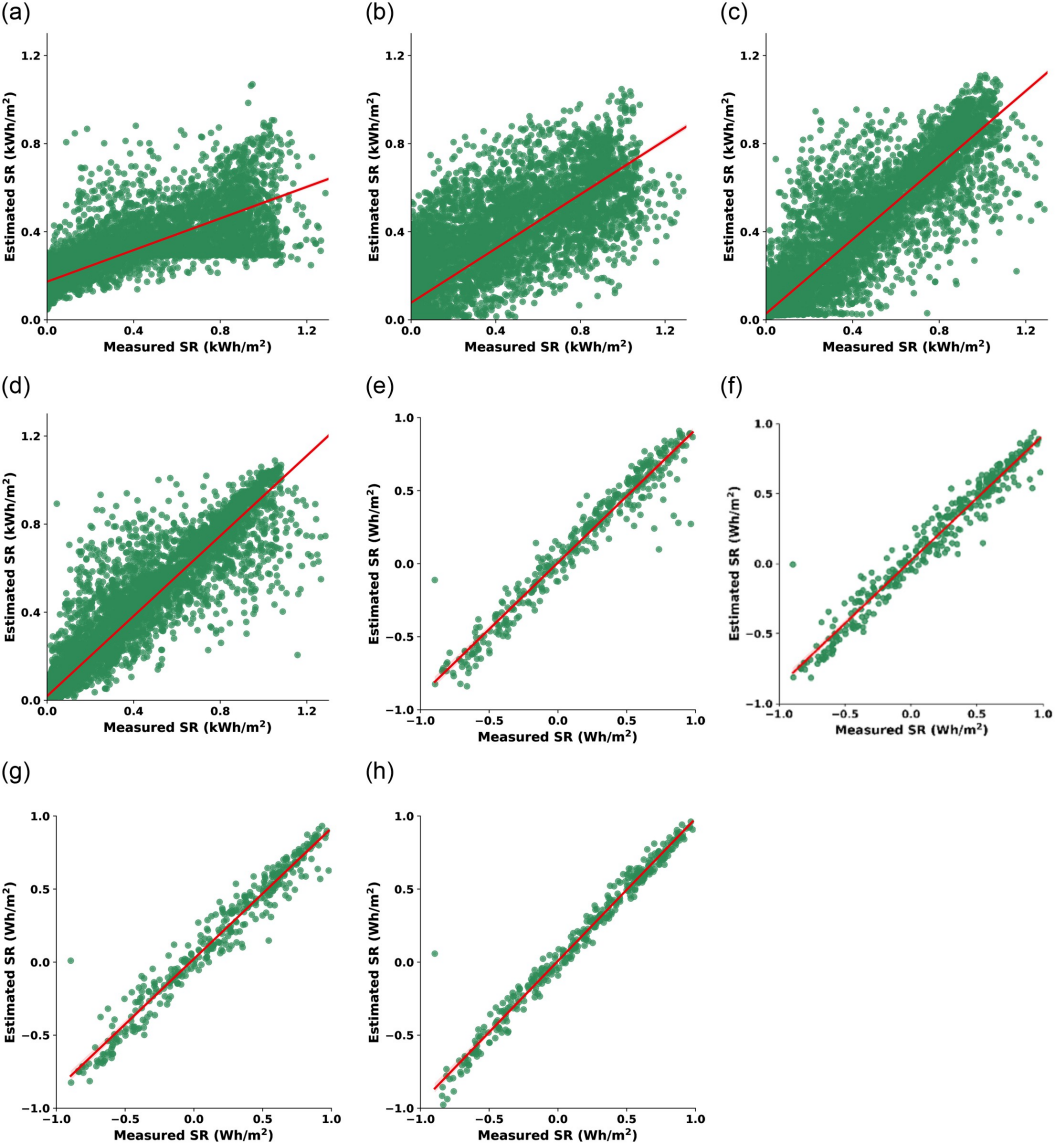
Convergence behavior of CO-optimized ML models using testing data of dataset-1 (first row, (a)-(d)) and dataset-2 (second row, (e)-(h)): CO-LR ((a) & (e)), CO-SVM ((b) & (f)), CO-ANN ((c) & (g)), and CO-RF ((d) & (h)).

In dataset-1, the SR values are measured in kilowatt-hours per square meter (kWh/m^2^). CO-LR has a maximum deviation from the ideal diagonal arrangement, indicating the worst performance among all models. CO-SVM exhibits a relatively higher spread of data points, indicating a wider dispersion of predictions. Conversely, CO-ANN shows a more clustered distribution of points, suggesting improved predictive consistency. However, the star performer in this dataset is CO-RF, which demonstrates the tightest clustering of points, signifying highly accurate SR predictions. In dataset-2, the SR values are in watt-hours per square meter (Wh/m^2^), and all three ML models exhibit commendable performance. The scatter plots for CO-SVM, CO-ANN, and CO-RF showcase strong positive correlations between predicted and actual SR values. In this case, CO-RF marginally outperforms the others, demonstrating a slightly tighter clustering of points, reaffirming its effectiveness in SR prediction even in datasets with different units. Fig 4 provides a set of scatter plots using testing data for different ML models applied to both SR datasets. In dataset-1’s, CO-SVM displays a relatively high spread of data points, suggesting some variability in its predictions. CO-ANN exhibits a more clustered distribution, indicating better predictive consistency. However, CO-RF outperforms both, showcasing the tightest clustering of points, signifying exceptionally accurate SR predictions on the testing data. In dataset-2’s, all three models (CO-SVM, CO-ANN, and CO-RF) continue to demonstrate strong positive correlations between their predictions and the actual SR values. While all models perform well, CO-RF exhibits a slightly tighter clustering of data points, reaffirming its effectiveness in SR prediction on the testing data.

In summary, Figs 3 and 4 serve as a visual confirmation of the models’ performance. The closer the data points align with the diagonal line, the more accurate the model’s predictions on unseen data. While CO-SVM, CO-ANN, and CO-RF exhibit their strengths, CO-RF consistently shines as a reliable choice for accurate SR prediction, particularly evident in the tight clustering of data in both datasets.

### CO-optimized ML model for temporal SR prediction

The SR prediction capability of different ML models is compared using training data and testing data. In dataset-1, UNIX time is used to arrange the records chronologically. Records corresponding to the first 1000 minutes of the data are used for visualization. In dataset-2, each record’s timestamp contains only the date; hence, no intraday temporal variation can be analysed. Instead, the first 100 records corresponding to different days in chronological order (not always consecutive days) are visualized.

**Fig 5:**
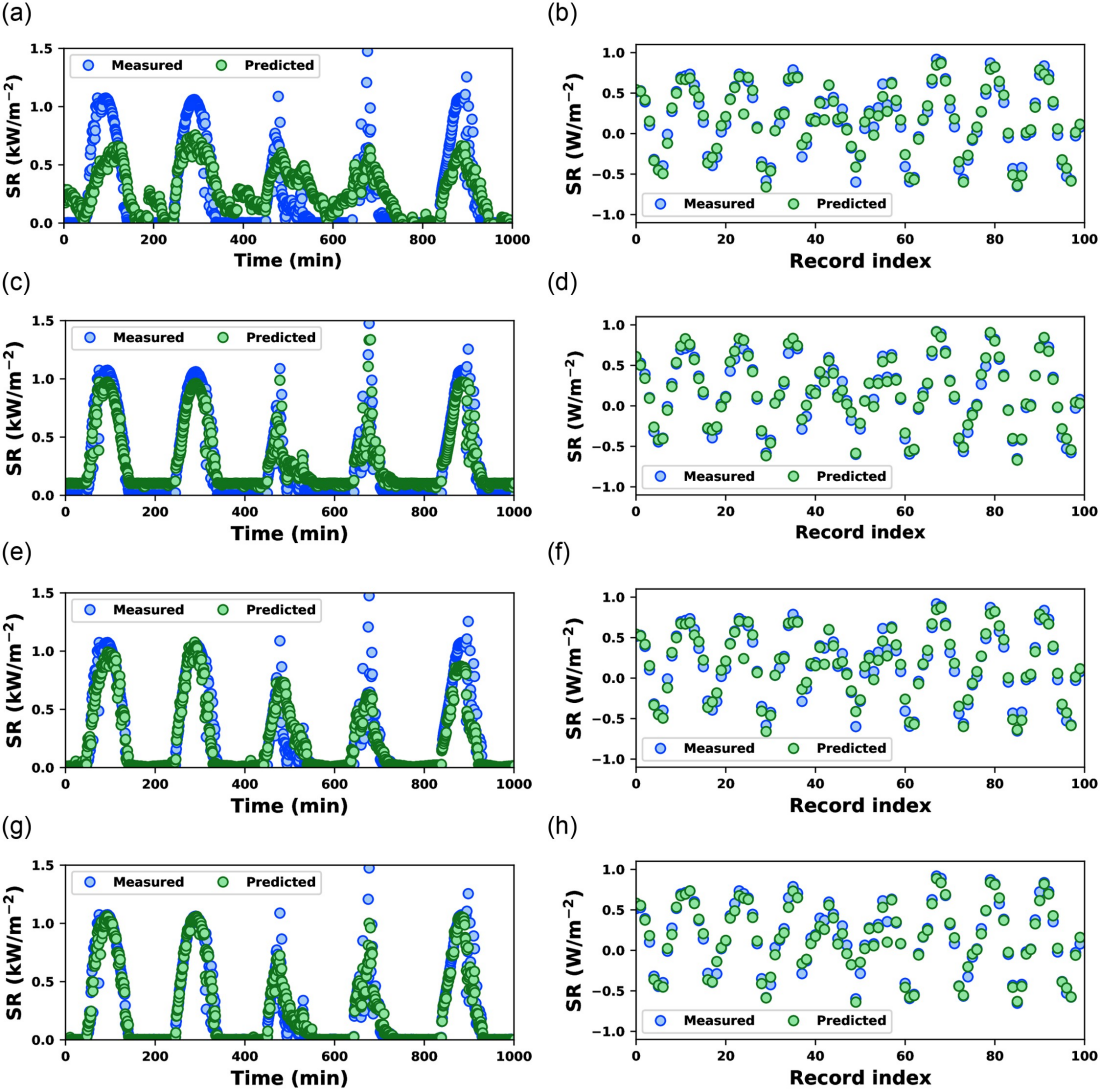
Measured and predicted solar radiation by CO-optimized ML models using training data of dataset-1 (first column) and dataset-2 (second column): CO-LR ((a) & (b)), CO-SVM ((c) & (d)), CO-ANN ((e) & (f)), and CO-RF ((g) & (h)).

The time series visualizations provide insights into how these models perform over time, aiding in assessing their suitability for SR prediction applications. Fig 5 uses a training subset of the datasets. Each column corresponds to a different dataset, and each row represents a different CO-optimized ML model. In dataset-1, it can be seen that CO-LR and CO-SVM missed several of the measured SR values. These models captured the basic periodic nature of the measured SR pattern but could not match the amplitude variation. CO-ANN, on the hand, performs better than CO-SVM in tracking the periodicity and amplitude variation. Finally, CO-RF almost exactly matches the measured SR values significantly better than the others.

**Fig 6:**
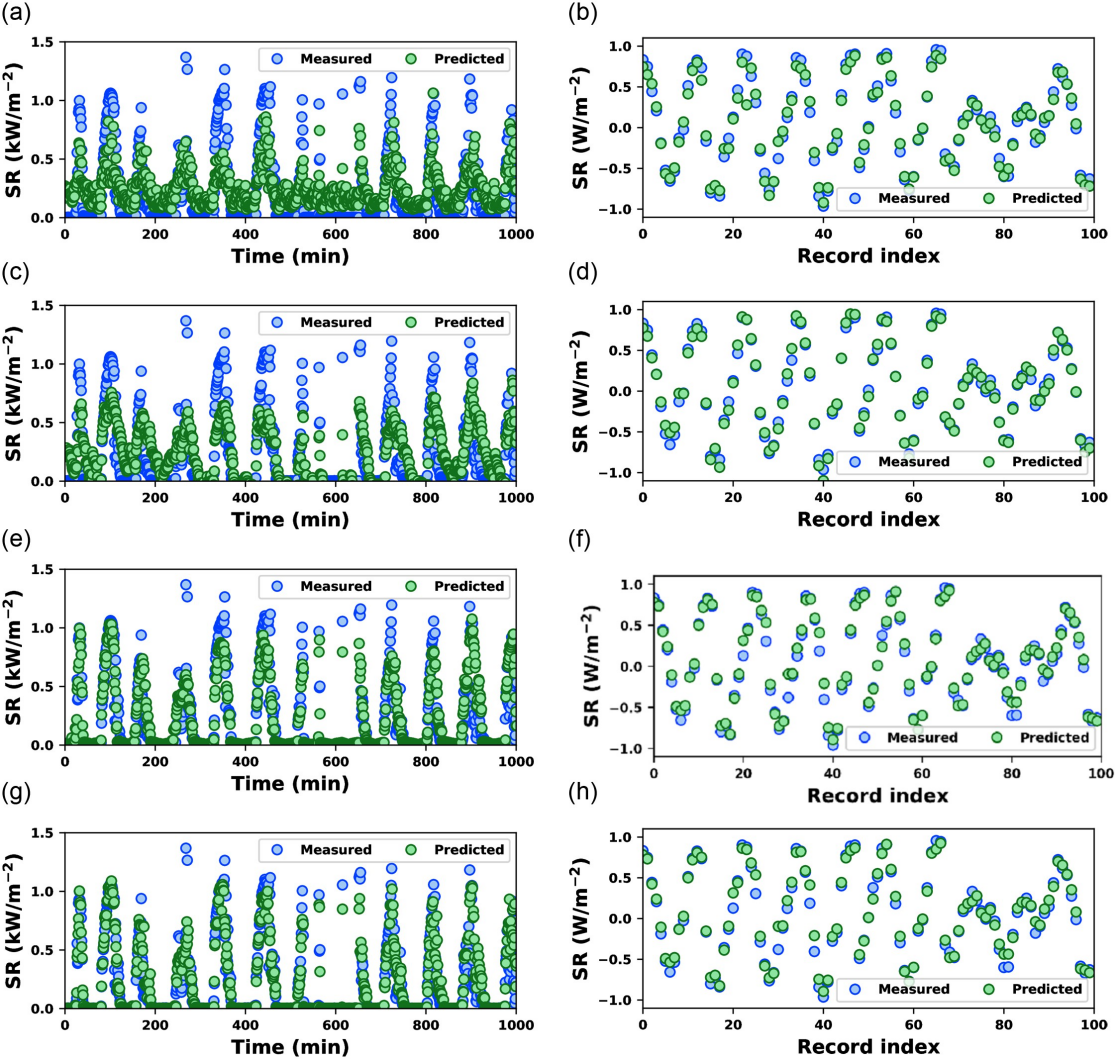
Measured and predicted solar radiation by CO-optimized ML models using testing data of dataset-1 (first column) and dataset-2 (second column): CO-LR ((a) & (b)), CO-SVM ((c) & (d)), CO-ANN ((e) & (f)), and CO-RF ((g) & (h)).

Fig 6 depicts a similar temporal relationship in the testing data. A similar observation can be noted in Fig 6. CO-LR and CO-SVM missed several SR values with higher amplitude correctly estimated by CO-ANN and CO-RF. Although CO-ANN and CO-RF visually miss almost identical SR values, the estimated SR values using CO-RF are much closer to measured SR values than the SR values estimated using CO-ANN. The CO-RF yielded better prediction results, and the temporal and amplitude variations in SR values performed best among the other examined models.

### Comparative analysis with existing work

Building on the advancements in machine learning models, the comparative analysis illustrates a clear trend toward increasing model complexity and predictive power. Table 6 compares existing literature on solar radiation prediction in different regions arranged chronologically. For instance, Marzo *et al*. [50] improved upon earlier methods with an artificial neural network (*R*^2^ = 0.8396), while Sharafati *et al*. [19] and Hassan *et al*. [51] adopted random forest and empirical predictive models, achieving impressive *R*^2^ values of 0.9312 and 0.9533, respectively. More sophisticated approaches emerged post-2020, such as deep learning techniques applied by Üstün *et al*. [52] and Ağbulut *et al*. [53], both of which demonstrated enhanced predictive accuracy (*R*^2^ = 0.9647 and 0.9378). The introduction of LSTM models, as seen in Ngoc-Lan Huynh *et al*. [54] (*R*^2^ = 0.9612), and Narvaez *et al*. [55] with random forest (*R*^2^ = 0.9654), showcased the potential of recurrent networks for time-series data.

**Table 6:** Comparative analysis with existing literature.

Reference	Year	Case study	Model	*R* ^2^
Olatomiwa *et al*. [56]	2015	Nigeria	Adaptive neuro-fuzzy model	0.6678
Marzo *et al*. [50]	2017	South Africa	ANN	0.8396
Hassan *et al*. [51]	2018	Egypt	Empirical predictive model	0.9533
Sharafati *et al*. [19]	2019	Burkina Faso	Random forest	0.9312
Guijo-Rubio *et al*. [57]	2020	Spain	Hybrid neural network	0.9728
Üstün *et al*. [52]	2020	Turkey	Deep learning	0.9647
Ağbulut *et al*. [53]	2021	Turkey	Deep learning	0.9378
Narvaez *et al*. [55]	2021	Colombia	Random forest	0.9654
Ngoc-Lan Huynh *et al*. [54]	2021	Vietnam	LTSM	0.9612
Rai *et al*. [58]	2021	Mauritania	Bi-LTSM	0.9805
Goliatt *et al*. [59]	2022	Burkina Faso	Multi-adaptive regression splines	0.9800
present work	2024	Soudi Arebia	CO with RF	0.9868

Finally, the present work leverages a CO-based random forest model, achieving the highest *R*^2^ (0.9868) reported to date, demonstrating the superior predictive capability of combining RF with CO techniques in the context of Saudi Arabia. This underscores the evolution of models towards increasing precision and adaptability across diverse geographical case studies.

## Conclusion and future work

SR prediction is well-studied for ML techniques to be utilized due to the capabilities of these models in providing reliable and robust predictions for SR values. This work introduced an efficient hybridization of the optimization algorithm and ML model for SR prediction. Two open-source datasets and three statistical metrics, MAE, MSE, and *R*^2^, are employed as evaluation measurements. Comparative analysis of five state-of-the-art optimization algorithms showed that CO is the most efficient for SR prediction. The LR, SVM, ANN, and RF standalone models are compared with hybrid models using CO-based feature selection. The experimental findings showed that CO-RF provides the best prediction performance compared to the other competitive methods with MAE of 0.0365, MSE of 0.0074, and *R*^2^ of 0.9251 on dataset-1 and MAE of 0.0469 of MSE of 0.0032, and *R*^2^ of 0.9868 on dataset-2. In future research, CO-RF can be applied to a large-scale IoT-based online monitoring system to predict SR based on real-time data from IoT sensors. Another possible research method is to use deep learning and recurrent architecture for SR prediction while powered by optimization algorithms.
